# The proportion of loss to follow-up from antiretroviral therapy (ART) and its association with age among adolescents living with HIV in sub-Saharan Africa: A systematic review and meta-analysis

**DOI:** 10.1371/journal.pone.0272906

**Published:** 2022-08-11

**Authors:** Cheru Tesema Leshargie, Daniel Demant, Sahai Burrowes, Jane Frawley

**Affiliations:** 1 Department of Public Health, College of Health Science, Debre Markos University, Debre Markos, Ethiopia; 2 School of Public Health, Faculty of Health, University of Technology Sydney, Ultimo, Australia; 3 School of Public Health and Social Work, Faculty of Health, Queensland University of Technology, Brisbane, Australia; 4 Public Health Program, College of Education and Health Sciences, Touro University California, Vallejo, CA, United States of America; Emory University School of Medicine, UNITED STATES

## Abstract

**Background:**

Human immunodeficiency virus (HIV) remains a global health threat, especially in developing countries. The successful scale-up of antiretroviral therapy (ART) programs to address this threat is hindered by a high proportion of patient loss to follow-up (LTFU). LTFU is associated with poor viral suppression and increased mortality. It is particularly acute among adolescents, who face unique adherence challenges. Although LTFU is a critical obstacle on the continuum of care for adolescents, few regional-level studies report the proportion of LTFU among adolescents receiving ART. Therefore, a systematic review and meta-analysis were conducted to estimate the pooled LTFU in ART programs among adolescents living with HIV in sub-Saharan Africa (SSA).

**Methods:**

We searched five databases (PubMed, Embase (Elsevier), PsycINFO, CINAHL, and Scopus) for articles published between 2005 and 2020 and reference lists of included articles. The PRISMA guidelines for systematic reviews were followed. A standardised checklist to extract data was used. Descriptive summaries were presented using narrative tables and figures. Heterogeneity within the included studies was examined using the Cochrane Q test statistics and I^2^ test. Random effect models were used to estimate the pooled prevalence of LTFU among ALHIV. We used Stata version 16 statistical software for our analysis.

**Results:**

Twenty-nine eligible studies (n = 285,564) were included. An estimated 15.07% (95% CI: 11.07, 19.07) of ALHIV were LTFU. Older adolescents (15–19 years old) were 43% (AOR = 0.57, 95% CI: 0.37, 0.87) more likely to be LTFU than younger (10–14 years old) adolescents. We find an insignificant relationship between gender and LTFU (AOR = 0.95, 95% CI: 0.87, 1.03). A subgroup analysis found that regional differences in the proportion of adolescent LTFU were not statistically significant. The trend analysis indicates an increasing proportion of adolescent LTFU over time.

**Conclusions and recommendations:**

The proportion of LTFU among HIV-positive adolescents in SSA seems higher than those reported in other regions. Older adolescents in the region are at an increased risk for LTFU than younger adolescents. These findings may help policymakers develop appropriate strategies to retain ALHIV in ART services. Such strategies could include community ART distribution points, appointment spacing, adherence clubs, continuous free access to ART, and community-based adherence support.

## Background

The prevalence of human immunodeficiency virus (HIV) infection continues to rise among adolescents in low-and middle-income countries [1, 2], particularly in sub-Saharan Africa (SSA) [3, 4], where 70% of people living with HIV (PLHIV) reside [5, 6]. In 2018, over 1.6 million adolescents in SSA lived with HIV; 190,000 adolescents were newly diagnosed with HIV, and 33,000 adolescents died from AIDS-related illnesses in the same year [7]. A relatively large proportion of adolescents living with HIV (ALHIV) was found in SSA compared to other regions [3, 8]. This was due primarily to high numbers of perinatal infections [8–10], the early age of sexual initiation [4, 9], and the scale-up of paediatric antiretroviral therapy (ART), which has resulted in increasing numbers of children living with HIV who survive to adolescence [3].

A high proportion of lost-to-follow patients increasingly challenges this successful scale-up of ART programs [11]. HIV treatment with ART requires consistent, long-term engagement with the health care system [12]. This is challenging for all PLHIV but may be especially difficult for adolescents who experience higher rates of loss to follow up (LTFU) in HIV treatment programs than younger children and adults [13–16].

There are several reasons for the relatively high rates of LTFU among adolescents. Firstly, while there have been steady improvements in HIV care and support for children and adults, in many settings, there remains significant room for improving diagnosis and treatment services for adolescents specifically as well as a recognised need for targeted policies and treatment guidelines for this population [17–19]. Secondly, a lack of coordination between paediatric and adult services may make navigating the health care system challenging for adolescents transitioning from paediatric to adult care [20]. Thirdly, the cognitive development and sensitivity to peer pressure that characterize adolescence may affect HIV treatment outcomes. There is evidence that adolescents living with chronic medical conditions such as HIV/AIDS often endure heightened social isolation as a result [16, 21]. For example, adolescents may skip HIV medicine prescriptions and be absent from health facilities to conceal their HIV status from their peers [22]. This peer pressure and isolation may lead to high-risk behaviour, including dropping out of HIV care [16].

Maintaining or improving retention (defined as a regular patient engagement with medical care at a health facility) in ART treatment services is a priority in HIV treatment policy and practice [23]. Low retention often leads to poor health outcomes, drug resistance, treatment failure, and patients being LTFU [24]. This, in turn, harms the sustainability and effectiveness of national HIV control and treatment efforts [17, 18].

Worldwide studies have identified several sociodemographic, clinical, and lifestyle factors associated with LTFU among ALHIV. Cohort data from the Asia-Pacific, the Caribbean, and Central and South American regions [25] have found LTFU associated with gender, age, and rural settings. Data from Thailand [26] have indicated that age and malnutrition have a significant association with LTFU.

In SSA, there are limited data on the treatment outcomes (i.e., death, viral suppression, and LTFU) of adolescents living with HIV [27]. Studies are also considerably varied in the proportion of adolescent LTFU they report [14, 28, 29], with estimates ranging from as low as 1.6% in South Africa [30] to as high as 32% in Uganda [31]. Such variation in the estimated proportion of adolescents LTFU in treatment programs makes it difficult for decision-makers to assess the problem and to develop appropriate and effective clinical and policy strategies in response. To address this lack of clarity, the current systematic review and meta-analysis aimed to estimate the pooled proportion of LTFU from ART services and its association with age among ALHIV in SSA. It also conducts a sub-group analysis to report regional LTFU estimates. The results obtained from this review provide basic background information for program planners and decision-makers designing regional and national strategies to reduce LTFU among adolescents. Furthermore, our review results could be used as baseline information for planning and evaluating national, regional, and international LTFU prevention strategies.

## Methods

We used the Preferred Reporting Items of Systematic Reviews and Meta-analysis (PRISMA) checklist (detailed in S1 File) to guide this systematic review. A systematic review and meta-analysis protocol was registered on the Prospero database (ID: CRD42020190193) [32].

### Inclusion and exclusion criteria

The study’s geographic scope comprises all 46 African countries in SSA [33]. All observational primary, peer-reviewed, English-language studies conducted in SSA that reported the proportion of LTFU from routine ART follow-up services and reported data on adolescents (using the WHO definition of those 10–19 years of age) were eligible for inclusion [34]. Studies published between January 1, 2005, and June 30, 2020, were included, as 2005 marks the year that the Joint United Nations Programme on HIV/AIDS (UNAIDS) announced a shift from small ART projects with short-term horizons to long-term, comprehensive strategies to manage the increase of HIV infections in the SSA region [35].

### Search strategy

The authors developed a search strategy (detailed in S2 File) cooperating with a specialist librarian at the University of Technology Sydney Library (UTS). Five databases were searched in June 2020: PubMed, Embase (Elsevier), PsycINFO, CINAHL, and Scopus. We also hand-searched the reference lists and citations of relevant studies identified from the database search. We searched for articles using Boolean operators and the following Medical Subject Headings

(MeSH) terms:

Line 1: "Antiretroviral therapy" OR "antiretroviral therapy" OR "Antiretroviral drugs" OR "antiretroviral drugs" OR "ART" OR "AIDS drugs" OR "Anti-AIDS drugs" OR "Anti-HIV drugs" AND "loss to follow-up" OR "lost to follow-up" OR "loss to follow up" OR "treatment outcome" OR "LTFU" AND
Line 2: "Teen*" OR "youth’’ OR "child*" OR "Adolescen*" AND
Line 3: "HIV" OR "Human immunodeficiency virus" OR "Human immunodeficiency infection" OR "AIDS" OR "acquired immunodeficiency syndrome" AND
Line 4: "all SSA countries"

### Outcome measure and definition of terms

The primary review outcome was the prevalence of LTFU from ART among ALHIV. The LTFU was defined as a patient who had missed three health facility visits or antiretroviral medication pick-ups [36–39] or had gone for more than 90 days without follow-up contact the last missed appointment and had not died or transferred out [39]. We directly extracted the total number of ALHIV and the number of adolescents LTFU from included studies. Then, the prevalence was computed by dividing the number of adolescents LTFU by the entire study population and multiplying by 100. The secondary analysis outcome was the effect of age on LTFU among ALHIV on ART follow-up.

### Heterogeneity across studies

Heterogeneity among included study effects and the pooled effect across studies was computed using the non-parametric Cochrane Q test [40]. Heterogeneity across studies was measured using I-squared (I^2^) test statistics [40]. A univariate meta-regression was used to identify the sources of heterogeneity between included studies.

### Articles screening and data extraction process

**Fig 1:**
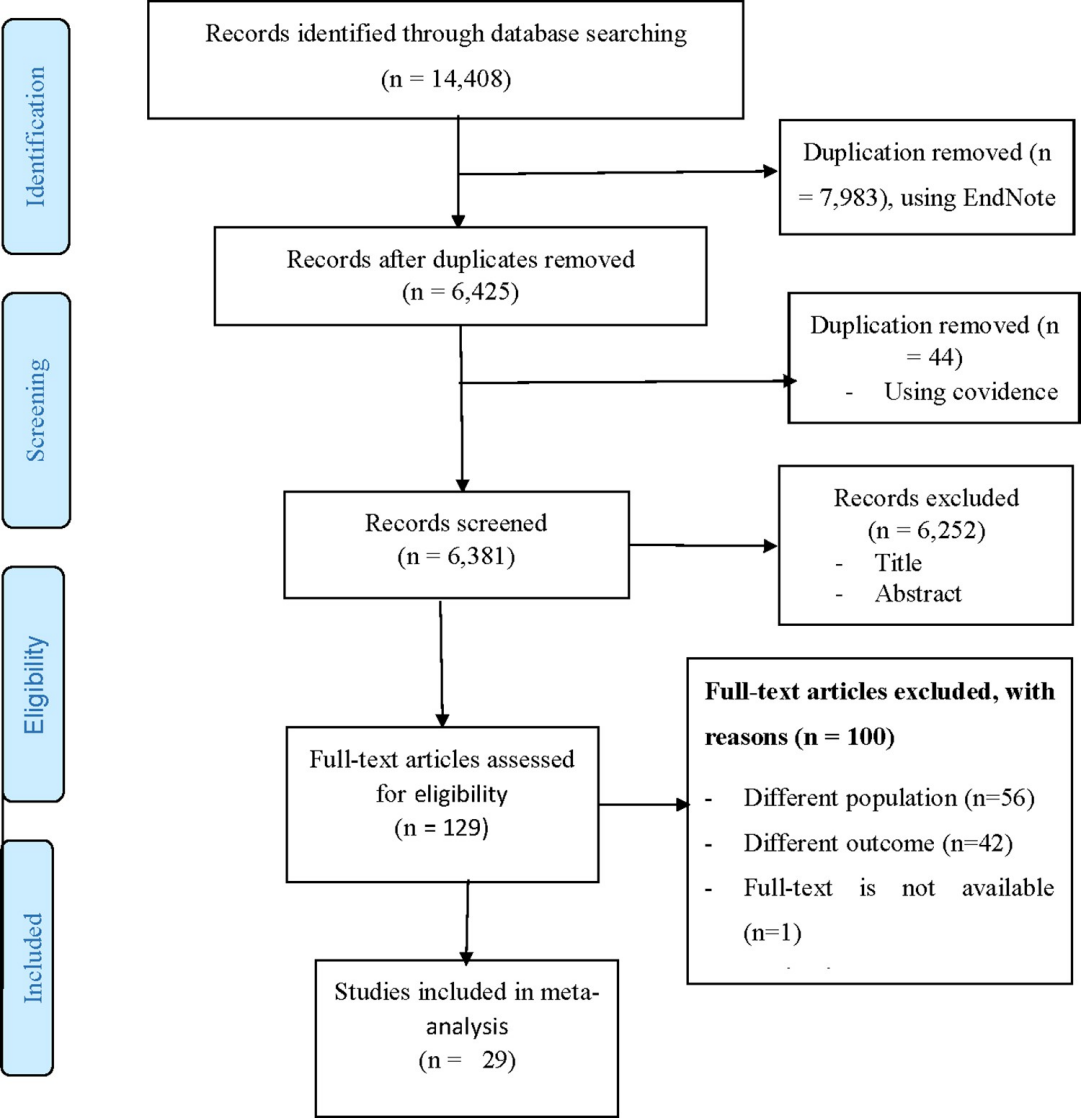
PRISMA flow diagram.

Fig 1 shows the PRISMA flow diagram for this systematic review. All search results (N = 14,408) were downloaded into the citation management software EndNote, version X^9^ (Thomson Reuters, London). Duplications (n = 7,983) were removed, and the remaining references (n = 6,425) were imported into Covidence (Veritas Health Innovative 2019) [41] for title screening, abstract and full-text review, and quality assessment. The Covidence software removed a further 44 duplicates. We screened 6,381 studies’ titles and abstracts, removing 6,252 irrelevant studies. A full-text assessment of the remaining 129 studies was performed. Two full-text articles were obtained from the primary author through email requests. One article was obtained through the UTS library. Full-text screening excluded 100 articles due to population differences (n = 56), differences in outcome of the study (n = 42), absence of full-text (n = 1) and study setting differences (n = 1). The final sample for the systematic review consisted of 29 studies.

The first author (CTL) extracted data from the original articles using a pre-piloted data extraction format in Microsoft™ Excel. The extraction format contained the first author, year of publication, study area/country, study design, sample size, outcome/LTFU, and adjusted hazard ratio/ adjusted odds ratio with 95% confidence intervals (CI) and age. Data on factors associated with LTFU were also extracted. Authors DD and JF checked extracted data for consistency.

### Quality assessment

We assessed the selected studies’ quality using the Newcastle Ottawa Scale (NOS), one of the most frequently used tools for evaluating the quality of observational studies in meta-analyses. The NOS evaluates three quality parameters: selection, comparability, and outcome, using eight items to measure the extent to which the research study addresses the possibility of bias in its method, design, and analysis [42]. Articles with a score ≥ 6 out of 10 were considered high quality (S3 and S4 Files). The first author (CLT) assessed the included studies’ methodological quality using the NOS assessment tool [43]. Author DD then conducted a second review. Inter-observer reliability was checked during regular project meetings to ensure consistency, with author JF resolving discrepancies between the two reviewers.

### Statistical analysis

**Fig 2:**
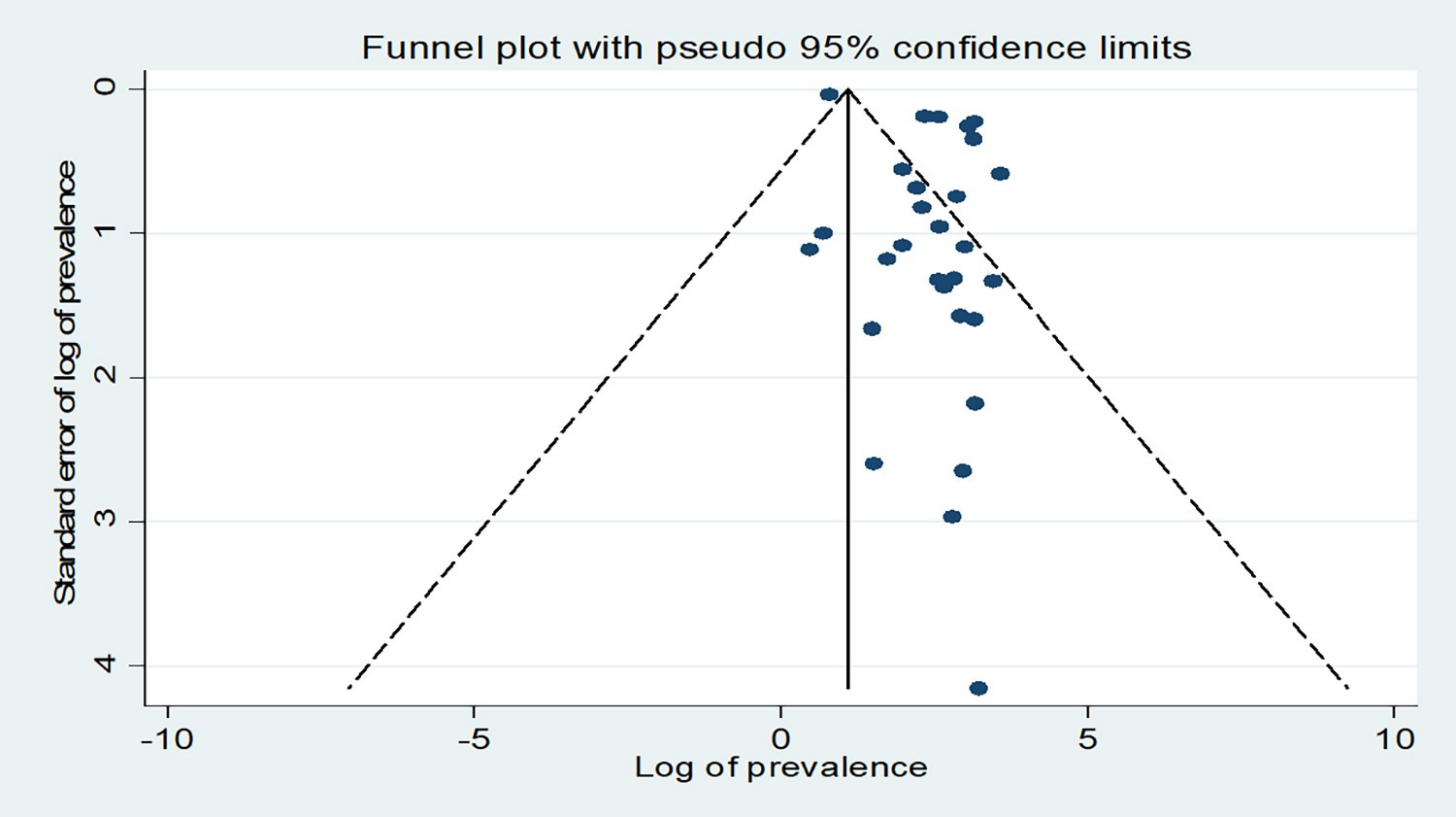
Funnel plot of the meta-analysis used to show a visual description of publication bias.

**Fig 3:**
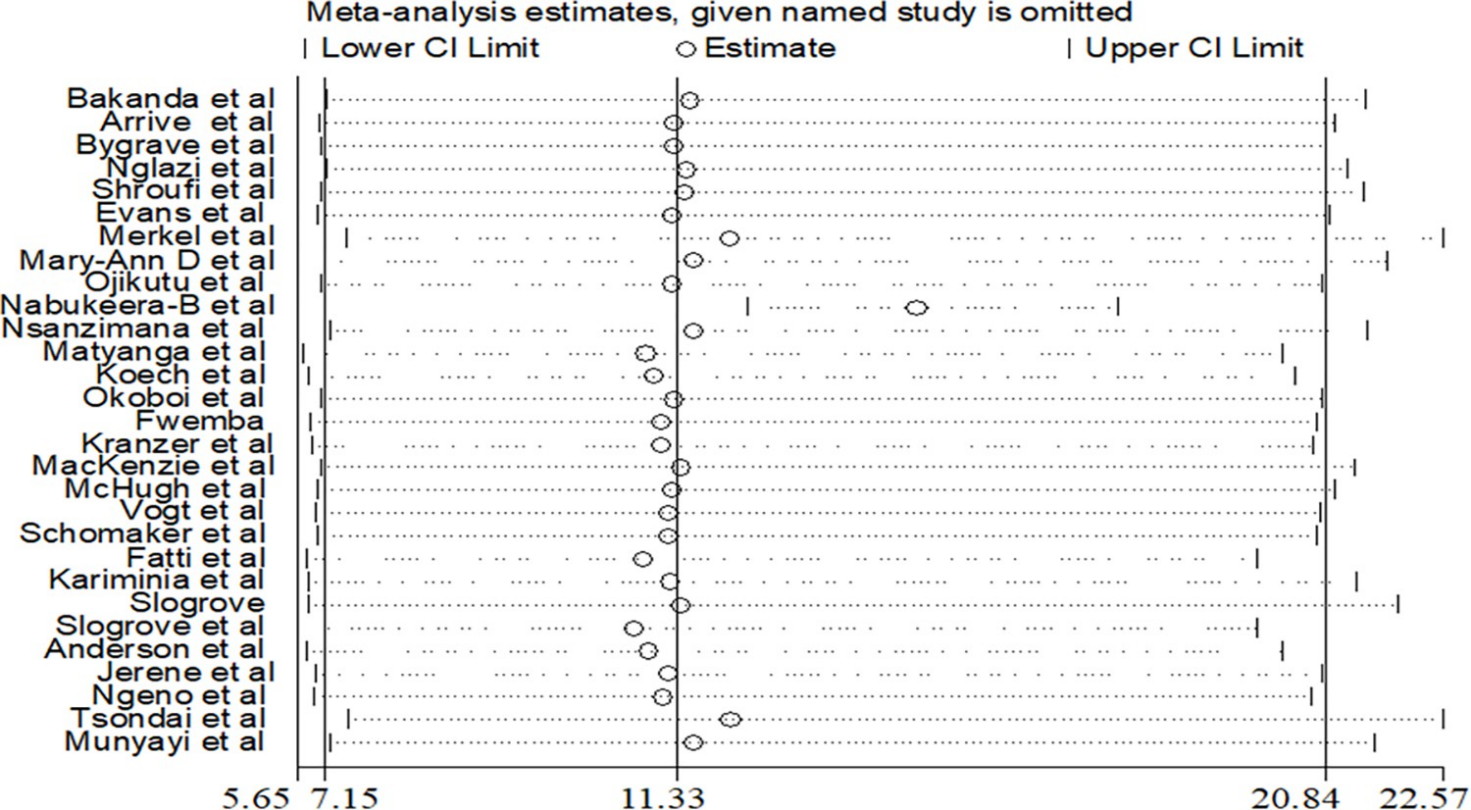
Sensitivity analysis of the pooled prevalence meta-analysis.

Extracted data was exported to Stata™ Version 16 for further analysis. A metan Stata command was used to compute the estimate of the pooled prevalence of LFTU. We used both funnel plot asymmetry and Egger rank correlation tests to check for the presence of publication bias. The funnel plot is a graphical visual representation of the trials plotted against their report effect size. The funnel plot visual inspection indicated asymmetry, which suggests publication bias (see Fig 2). The Egger weighted regression method was applied to assess publication bias with a p-value <0.05 demonstrating the presence of significant publication bias [44]. The Egger regression test results (p = 0.001) confirmed the publication bias in the studies under review. The DerSimonian and Laird method was used to produce a random-effects meta-analysis. A sensitivity analysis was performed to assess a study that influenced the overall pooled estimate (see Fig 3). Subgroup analyses were performed by geographical region and study design to minimise the observed random variation between the primary studies. Finally, the effect of adolescent age on LTFU was examined, and the result was described using Adjusted Odds Ratios (AOR). Effect sizes were presented as adjusted odds ratios (AOR) with 95% CIs. The meta-regression analysis considered the odds of LTFU among young adolescents (10–14 years old) compared with older adolescents (15–19 years old) who were in the reference (control) category.

## Results

### Characteristics of the included primary articles

Twenty-nine studies (see Table 1) were included in the systematic review, with a combined sample size of 285,564 adolescents who had initiated ART. The smallest sample size was 65 [13], which was obtained from a South African study. The largest sample size (129,405) was obtained from a study conducted in Rwanda [45]. The majority (n = 23, 79.3%) of the included studies were retrospective cohort studies/analyses [14, 16, 28, 30, 31, 46–63]. We also included three (10.3%) prospective cohort studies [15, 64, 65], one (3.4%) case-controlled study [66], one (3.4%) comparative cross-sectional study [67], and one (3.4%) [45] cross-sectional analysis.

**Table 1:** Descriptive summary of 29 studies included in the meta-analysis of LTFU among HIV-positive adolescents (10–19 years old) on ART in SSA.

Author	Publication year	region	Study design	Sample size	Event of LTFU	Prevalence of LTFU (%)	Quality assessment
Bakanda et al. [47]	2011	Uganda	Retrospective cohort study	575	42	7.3	High (7)
Arrive et al. [46]	2012	West Africa (Cote d’Ivoire, Mali, and Senegal)	Retrospective cohort study	650	85	13.1	High (7)
Bygrave et al. [15]	2012	Zimbabwe	Prospective cohort study	157	26	16.6	High (7)
Nglazi et al. [13]	2012	South Africa	Prospective cohort study	65	3	4.6	High (7)
Shroufi et al. [59]	2013	Zimbabwe	Retrospective cohort study	1,776	164	9.2	Low (5)
Evans et al. [14]	2013	South Africa	Retrospective cohort study	651	94	14.4	High (7)
Merkel et al. [53]	2013	Rwanda	Retrospective cohort study	196	4	2.0	High (7)
Mary-Ann Davies et al. [51]	2014	Southern Africa (Malawi, South Africa, and Zimbabwe)	Retrospective cohort study	2,161	158	7.3	High (6)
Ojikutu et al. [57]	2014	Nigeria	Retrospective cohort study	225	44	19.6	High (7)
Nabukeera-Barungi et al. [55]	2015	Uganda	Retrospective cohort study	156	7	4.5	High (8)
Nsanzimana et al. [45]	2015	Rwanda	Cross-sectional analysis	129,405	2,847	2.2	High (7)
Matyanga et al. [52]	2016	Zimbabwe	Retrospective cohort study	110	28	25.5	High (7)
Koech et al. [50]	2014	Kenya	Retrospective cohort study	14,840	3,478	23.4	High (8)
Okoboi et al. [31]	2016	Uganda	Retrospective cohort study	1,228	393	32	High (9)
Fwemba & Musonda [67]	2017	Zambia	Comparative cross-sectional	1,334‬	268	20.1	High (9)
Kranzer et al. [16]	2017	Zimbabwe	Retrospective cohort study	1,260	167	13.3	High (9)
MacKenzie et al. [66]	2017	Malawi	Case-control	617	116	18.8	High (8)
McHugh et al. [64]	2017	Zimbabwe	Prospective cohort study	385	92	24	High (7)
Vogt et al. [63]	2017	Zimbabwe	Retrospective cohort study	1,260	167	13.3	High (6)
Schomaker et al. [58]	2017	Southern Africa and West Africa	Retrospective cohort study	2,618	467	17.8	Low (5)
Fatti et al. [48]	2018	South Africa	Retrospective cohort study	6,706	2,414	36	High (7)
Kariminia et al. [49]	2018	Sub-Saharan Africa (Central, East, Southern, West)	Retrospective cohort study	35,494	8,448	23.8	High (8)
Slogrove [60]	2018	Low, middle, and upper-income sub-Saharan Africa country	Retrospective cohort study	90,888	10,725	11.8	High (7)
Slogrove et al. [61]	2018	Sub-Saharan Africa	Retrospective cohort study	30,168	3,982	13.2	High (8)
Anderson et al. [30]	2019	South Africa	Retrospective cohort study	127	2	1.6	High (7)
Jerene et al. [28]	2019	Ethiopia	Retrospective cohort study	816	138	16.9	High (7)
Ngeno et al. [56]	2019	Kenya	Retrospective cohort study	710	168	23.7	High (8)
Tsondai et al. [62]	2019	South Africa	Retrospective cohort study	25,401	5,436	21.4	Low (5)
Munyayi et al. [54]	2020	Namibia	Retrospective cohort study	385	22	5.7	High (8)

Six studies [15, 16, 52, 59, 63, 64] were conducted in Zimbabwe; five [13, 14, 30, 48, 62] in South Africa; three [15, 31, 55] in Uganda; two in Kenya [50, 56] and Rwanda [45, 53] each, and one study was conducted in Ethiopia [28], Zambia [67], Malawi [66], Namibia [54], and Nigeria [57]. The remaining five (n = 5) studies took place in more than one sub-Saharan Africa country [46, 49, 51, 58, 60] (Table 1).

### Quality assessment

Three authors (CT, DD, and JF) assessed the quality of included studies using the New Ottawa Scale (NOS). The NOS quality scores for the included studies ranged from five to nine, with a mean score of 7.52 (SD: 1.15). Based on the NOS, the majority, 26 (89.7%) of the included studies, had good quality. The remaining three (10.3%) included articles were rated "low", with scores below the cut-off point (Table 2).

**Table 2:** Describe articles rated lower quality using Newcastle Ottawa Scale (NOS) rating scale.

Studies	Description of the quality status of included studies categorised as poor quality
Nglazle et al.	• The response rate (i.e., the proportion of the study sample completing the study and providing outcome data) is inadequate.
Nsanzimana et al.	• The sample size is not determined using the recommended assumptions • The response rate (i.e., the proportion of the study sample completing the study and providing outcome data) is inadequate. • Quality management is not adequately described, no training for data collectors is given
Marry-Ann et al.	• Eligibility criteria are not clearly defined • lack of description of the derivation of the exposed group • An exciting variable is not clearly defined

### Meta-analysis

**Fig 4:**
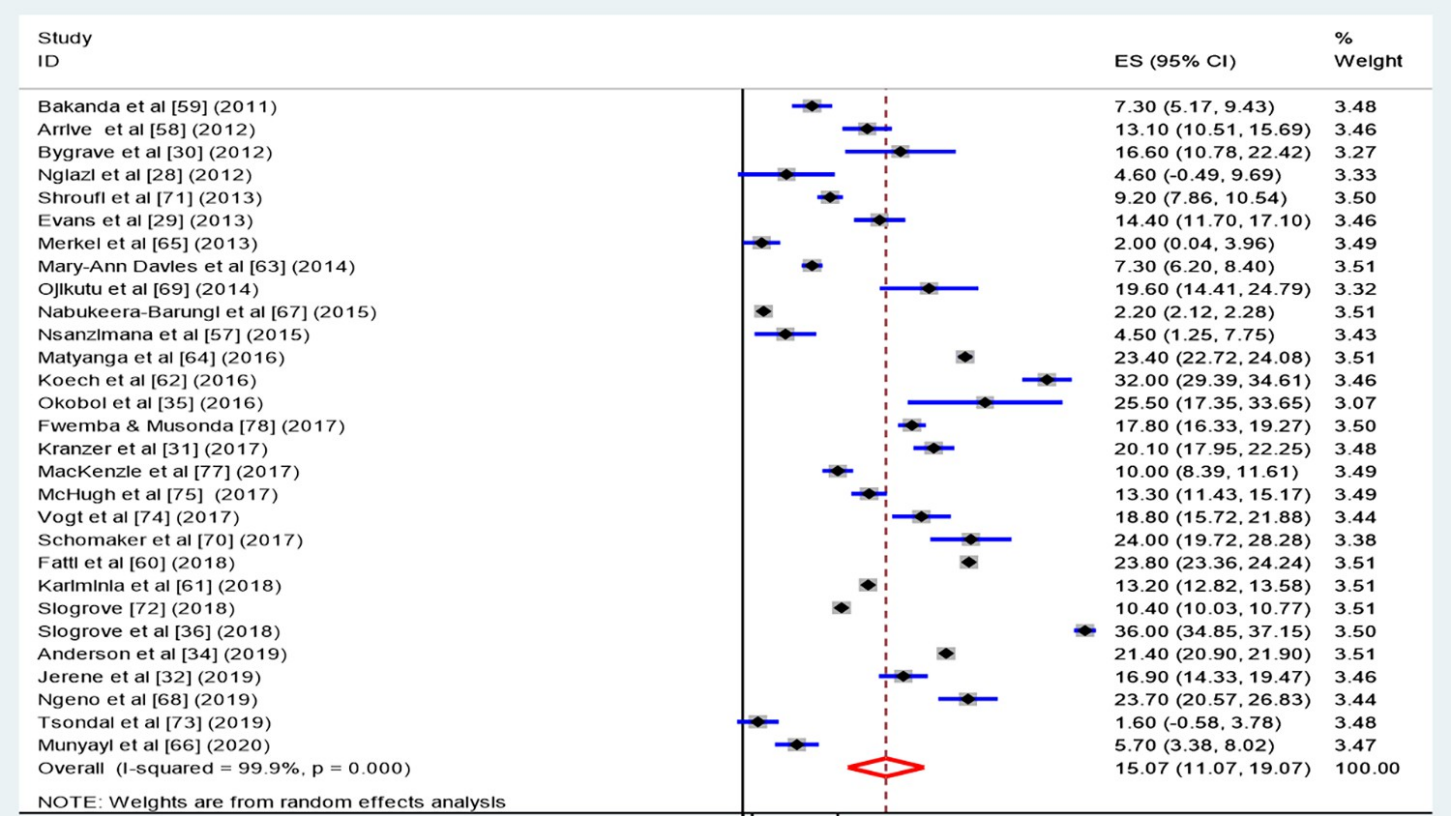
Pooled prevalence of estimated loss to follow-up among adolescents living with HIV and on ART follow-up in SSA.

The pooled estimated LTFU (see Fig 4) computed from the 29 included studies was 15.07% (95% CI: 11.07, 19.07). The lowest (1.6%) and highest (36%) proportions of LTFU reported were both found in studies conducted in South Africa [30, 48]. The second-highest proportion of LTFU (32%) was reported by a study conducted in Uganda in 2016 [68].

The Cochrane-Q test (p < 0.001) and I^2^ test statistics (I^2^ = 99.9%) indicated the existence of heterogeneity between the included studies. A random-effects model was applied to estimate the effect size of LTFU among adolescents living with HIV.

### Subgroup analyses

**Fig 5:**
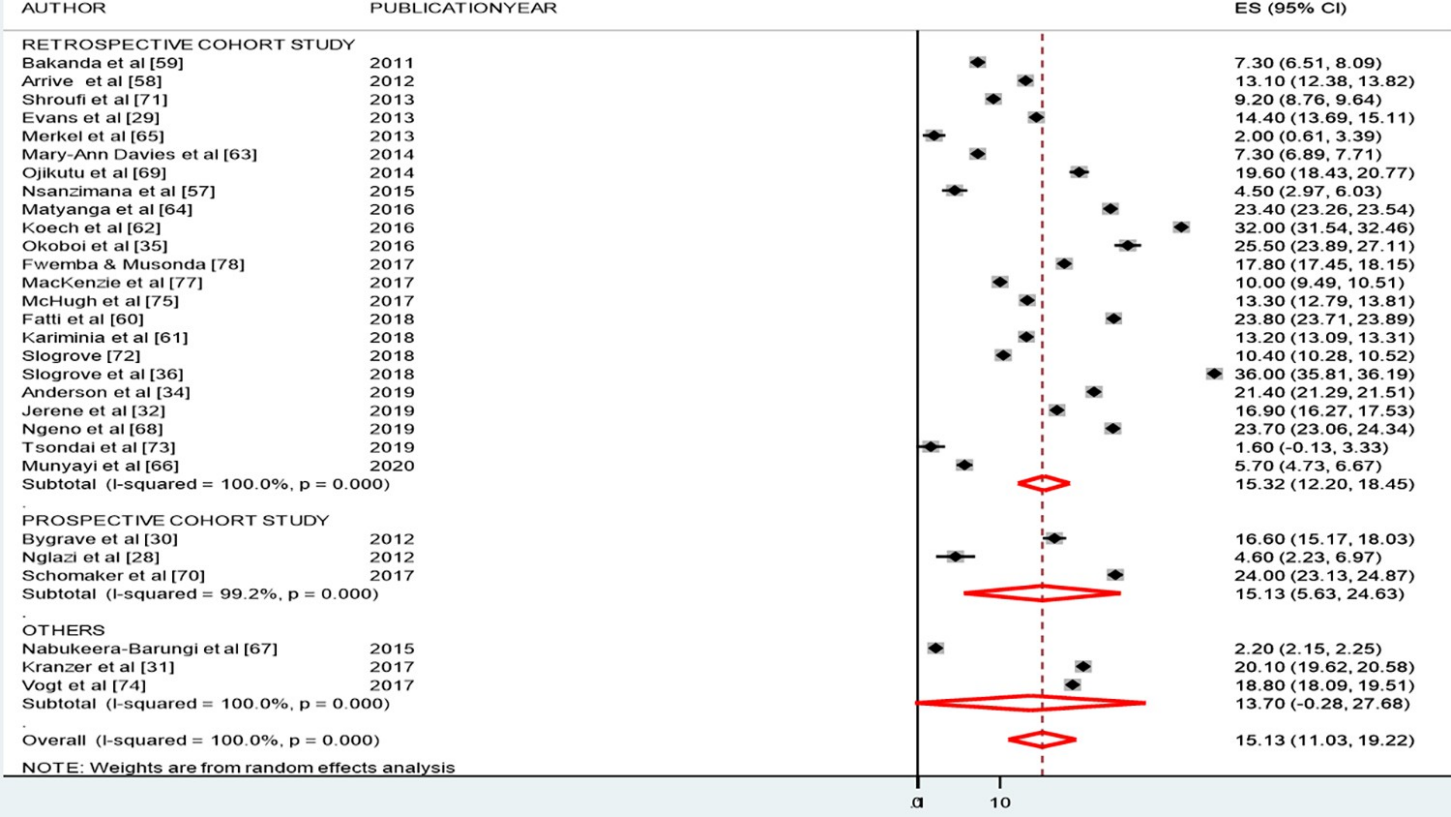
Subgroup analyses by study design on loss to follow-up among adolescents living with HIV and on ART follow-up in SSA.

**Fig 6:**
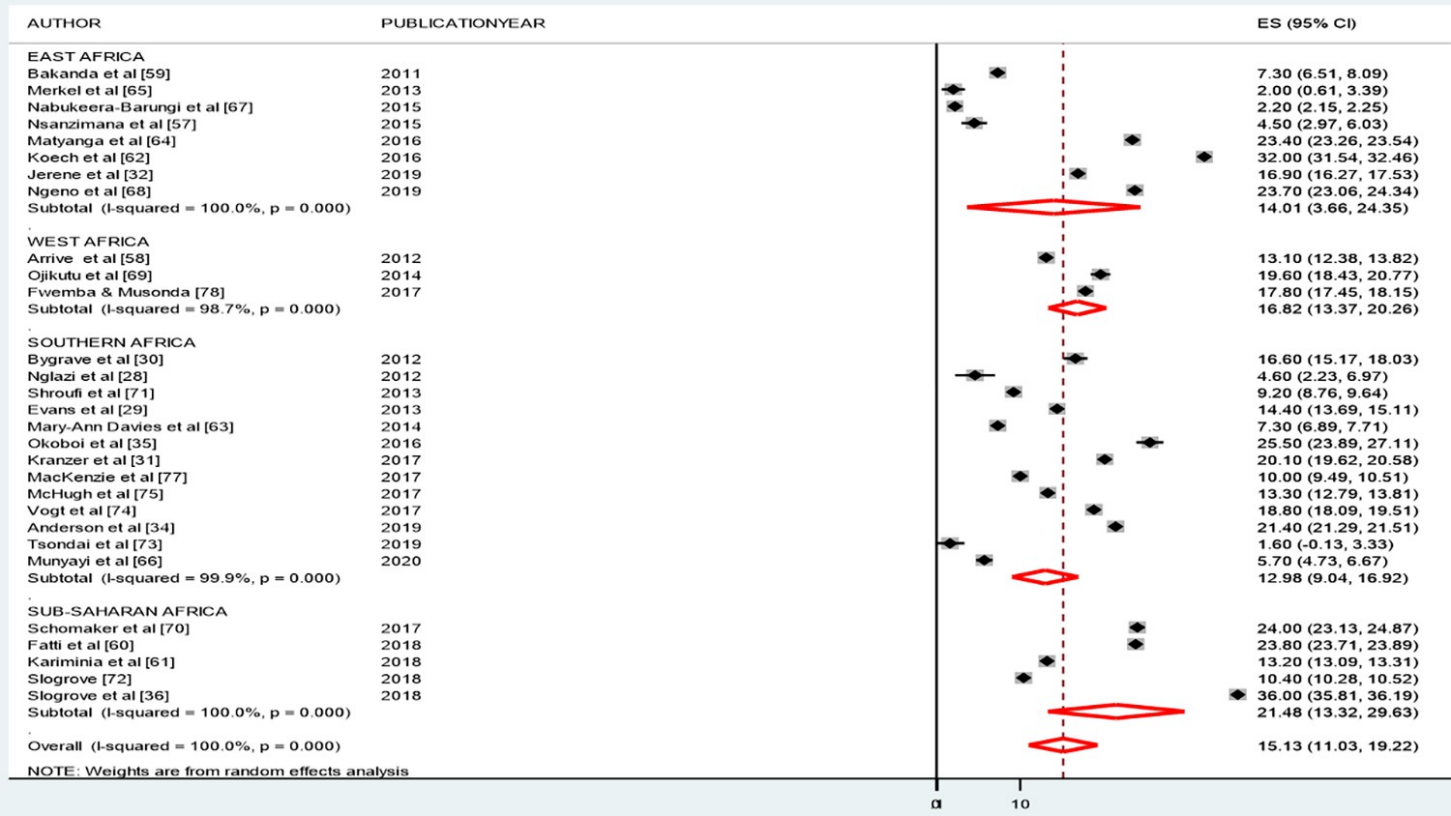
Subgroup analyses by the broad categories of across national countries on loss to follow-up among adolescents living with HIV and on ART follow-up in SSA.

We also conducted subgroup analyses by study design (see Fig 5) and SSA geographical regions (see Fig 6). The results of our subgroup analysis by study design suggested a similar proportion of LFTU across all study designs. The proportion of LTFU was 15.21% (95% CI 12.11, 18.31), for retrospective cohort studies; 15.11% (95% CI: 3.37, 26.86) for prospective cohort studies; and 13.66% (95% CI: 0.35, 27.66) for other study designs (i.e., comparative cross-sectional, case-control and cross-sectional) (see Fig 5).

In the subgroup analysis assessed by region; the proportion of adolescents LTFU was 12.98% (95% CI: 9.04, 16.92) for Southern African studies; 14.01% (3.66, 24.35) for East Africa; and 16.82% (13.37, 20.26) for West Africa and 21.48% (95% CI: 13.32, 29.69) for SSA-wide studies (see Fig 6).

### Factors associated with LTFU

**Fig 7:**
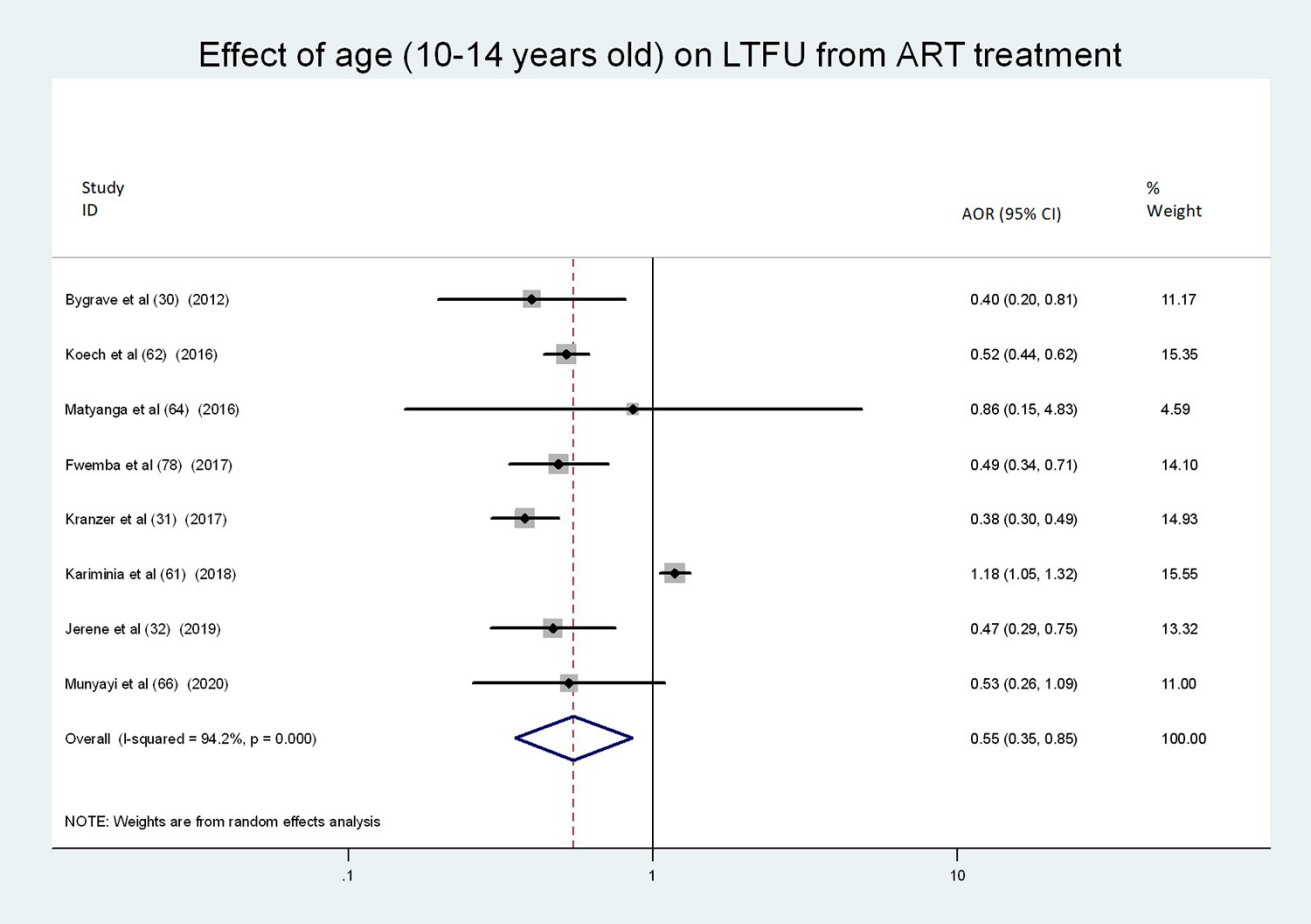
Forest plot showing the association of age and LTFU in ART treatment.

Eight of the included studies examined factors associated with LTFU using multivariable logistic regression. The factors that were considered in the studies are outlined in Table 3. The proportion of LTFU among adolescents was significantly correlated with age, time of ART initiation, disclosure status, CD4+ cell count, urban/rural residence, and baseline WHO stage. Younger ALHIV (10–14 years old) were 43% less likely to be LTFU (AOR = 0.57, 95% CI: 0.37, 0.87) compared to older adolescents (age 15–19 years old) [15, 16, 28, 49, 50, 52, 54, 67] (see Fig 7).

**Table 3:** Descriptive summary of 8 included studies on the factors associated with LTFU among HIV-positive adolescents between 2005 and 2020.

Articles	Publication year	Study design	Sample size	Prevalence of LTFU (%)	Adjusted confounder for predictors of lost to follow-up
Munyayi et al. [54]	2020	Retrospective cohort study	385	5.7	Sex, age, CD4 count, Hgb at ART initiation, WHO clinical staging, BMI, kg/m2
Jerene et al. [28]	2019	Retrospective cohort study	816	16.9	Age group, residence, CD4 at ART initiation (count/ml), Hgb at ART initiation, sex
Kariminia et al. [49]	2018	Retrospective cohort study	35,494	23.8	Sex, age, CD4 count, cells/mm3, WHO clinical staging, BMI, kg/m2, TB at ART initiation, ART regimen at Initiation, HIV status disclosed
Kranzer et al. [16]	2017	Retrospective cohort study	1,260	13.3	Sex, baseline age, current age, and time on ART
Fwemba & Musonda [67]	2017	Comparative cross-sectional	1,334‬	20.1	Age, ART regimen, WHO Stage, Time on ARV (months), sex, Baseline CD4 count, cell/mm3, Baseline hemoglobin
Matyanga et al. [52]	2016	Retrospective cohort study	110	25.5	Baseline age, CD4 count, Sex, WHO clinical staging, BMI, kg/m2, TB at ART initiation, HIV status disclose, ART regimen at initiation
Koech et al. [50]	2014	Retrospective cohort study	14,840	23.4	Age, CD4 cell count, WHO clinical stage, sex
Bygrave et al. [15]	2012	Prospective cohort study	157	16.6	Age, CD4 counts, sex, year of Initiation, Time on ART (days),

Not knowing one’s HIV status dramatically increased the odds of LTFU among adolescents (AOR = 23.7, 95% CI: 17.9, 30.8, p<0.05) [14, 27, 56]. Late ART initiation was also significantly associated with an increase in the proportion of adolescents LTFU (AOR = 8.72, 95% CI: 5.85, 13.02, p<0.05) [28, 30, 49, 50, 69–71]. Adolescents with lower baseline CD4+ cell counts were more likely to be LTFU than adolescents with higher baseline CD4+ cell counts (AOR = 0.12, 95% CI: 0.10, 0.15, p<0.05) [25]. LTFU was significantly higher (AOR = 1.40, 95% CI: 1.13, 1.75, p<0.05) among adolescents from rural areas than those from urban areas [25]. Finally, adolescents in WHO Clinical Stages III and IV experienced higher LTFU than those in Stages I and II (AOR = 2.51, 95% CI: 1.90–3.13, p<0.05) [50, 70].

**Fig 8:**
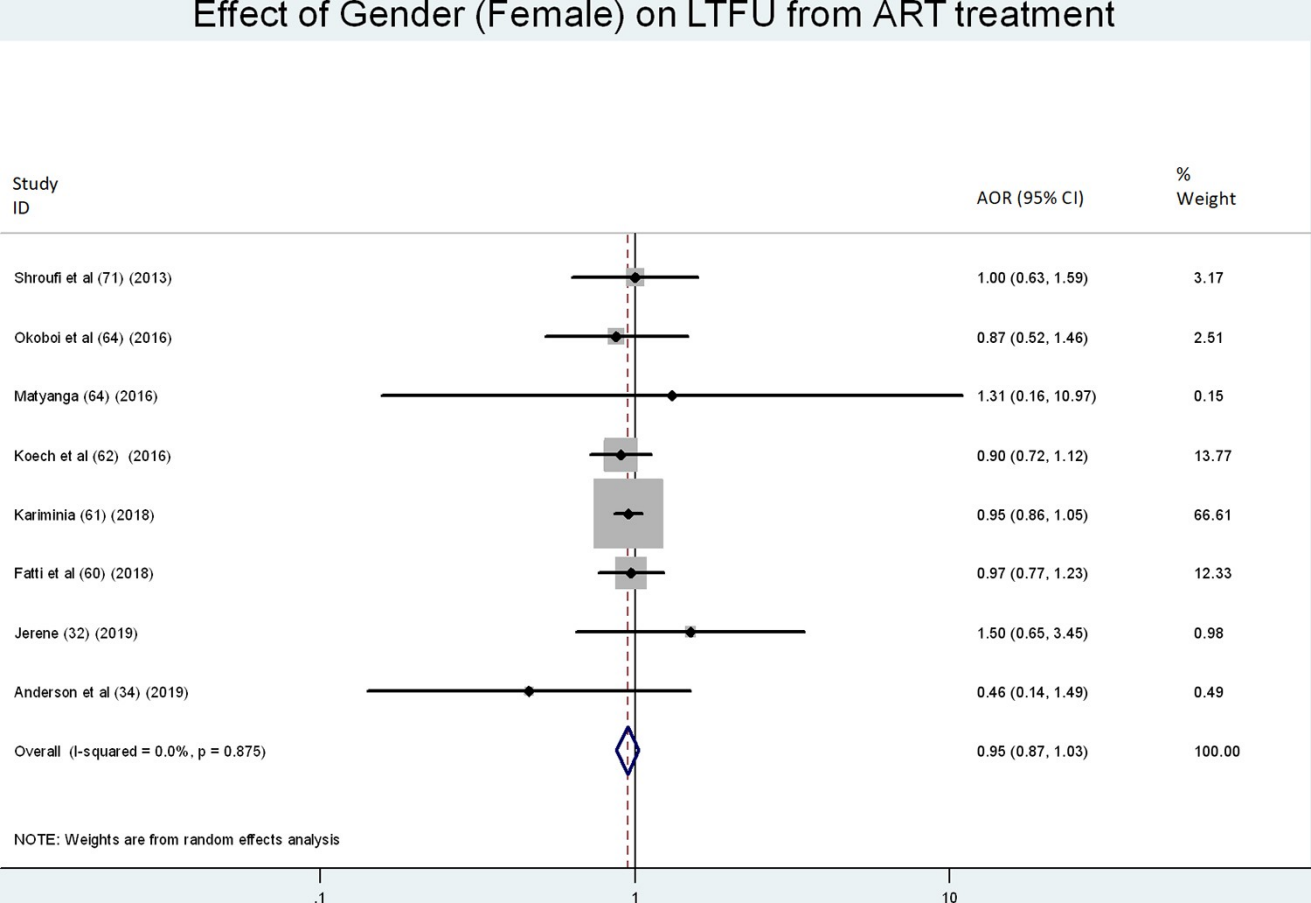
Forest plot showing the association of gender and LTFU in ART treatment.

Eight [28, 30, 31, 48–50, 52, 59] of our included studies assessed the effect of gender on LTFU among ALHIV, but our analysis found no significant relationship between gender and LTFU in this population (AOR = 0.95, 95% CI: 0.87, 1.03) (Fig 8).

### Trend of LTFU

**Fig 9:**
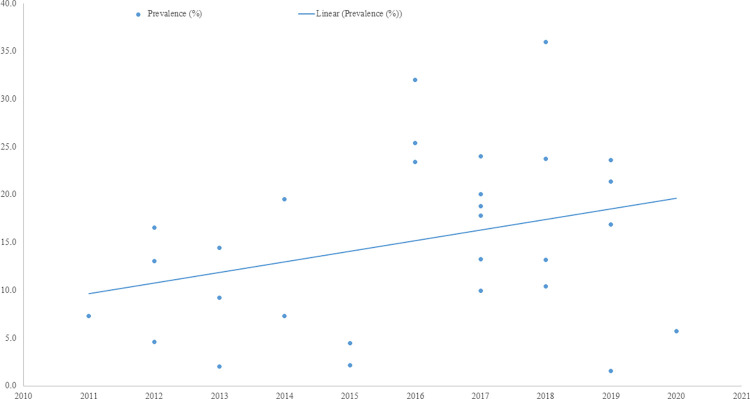
The time trend of LTFU among HIV positive adolescents in sub-Saharan Africa from 2005 to 2020.

A time-trend analysis of LTFU among ALHIV in SSA was estimated from the studies for the 2005 to 2020 time period. The analysis indicated that the proportion of LTFU increased from 2011 to 2020 (see Fig 9).

## Discussion

This systematic review and meta-analysis aimed to estimate the overall proportion of LTFU among ALHIV and its associations with age. To our knowledge, this systematic review and meta-analysis are the first in this region, focusing on LTFU in this population. LTFU is one of the main threats to patient retention and may persist even after ART services’ scale-up [72].

We estimate that the pooled proportion of LTFU among adolescents in SSA was 15.07% (95% CI: 11.07, 19.07). This estimated prevalence is higher than those found previously in Europe (6.1%), North America (8.9%), South and Southeast Asia (7.1%), South America and the Caribbean (4.8%) [61, 73], and India (9.2%) [73].

The differences between our LTFU estimate and those found in other regions may be due to more fragile healthcare systems and a higher proportion of the population being of lower socioeconomic status in SSA. ART services are usually offered free of charge in national treatment programs in SSA; however, financially disadvantaged patients may still face considerable difficulties accessing care due to transportation costs, income loss from time away from work, and other logistical challenges [74–76]. In addition to patient-level reasons for high LTFU in SSA, it must be noted that high LTFU in ART programs is usually a symptom of more considerable health system weakness [77], specifically a lack of resources required to offer the support, counselling, and outreach needed for patient retention.

The subgroup analysis by study setting indicates that variation between SSA regions was not statistically significant. The analysis found that the proportion of LTFU from ART programs was 14.01% (95% CI: 3.66, 24.35) in East Africa, 16.82% (13.37, 20.26) in West Africa, 12.98% (9.04, 16.92) in Southern Africa, and 21.48% (13.32, 29.63) in other SSA regions. A similar socioeconomic environment might explain the statistical insignificance of the variation in the proportion of LTFU between SSA regions.

Our time trend analysis of LTFU among ALHIV in SSA between 2005 and 2020 showed a linear trend with LTFU increasing each successive year after 2011. This might be explained by improvements in the quality of ART programs’ record-keeping and data management systems over time, potentially leading to a more accurate reporting of LTFU in this population [78]. However, more precise reporting could just as easily lead to decreasing LTFU as people formerly misclassified as LTFU are correctly accounted for [79]. Studies have found that poor data management and inaccurate case definitions account for about 36% of the treatment outcomes that are wrongly classified [79]. Changing definitions of LTFU over time and across different countries may also explain increasing levels of LTFU. For example, the recommendation that more than 180 days of non-contact be used as the definition for LTFU was only made in 2011 [80], but many current ART treatment guidelines still define LTFU as 90 days without contact [36–39]. However, if countries have increasingly adopted this more stringent definition, we would expect to see levels of LTFU falling over time, not increasing. Rather than record-keeping or definition changes, growing socioeconomic pressures and demographic changes may have made retention more difficult in the region. As the history of the pre-treatment HIV/AIDS pandemic fades for younger cohorts, attitudes about the virus and knowledge of treatment may also be changing [5].

The secondary objective of this study was to identify associations between LTFU and age in this population. We found that older adolescents had higher rates of LTFU. This age effect seems to be found consistently in low- and high-income settings. For example, a study conducted in India on the same population reported that age affected treatment outcomes such as LTFU [73], as did cohort data from the Asia-Pacific region, the Caribbean, and Central and South America [25]. However, at least one study conducted in Thailand [26] reported that LTFU was higher among younger ALHIV.

There are several reasons why older adolescents may experience more difficulties remaining in ART treatment programs than their younger peers. Firstly, as adolescents’ age increases, awareness about HIV stigma may also increase and negatively affect their willingness to use ART services or disclose their HIV status [81]. Secondly, in the later stage, 15–19 years of age, adolescents are increasingly sexually active, leading to apprehensions about inadvertent partner disclosure if seen receiving HIV care [82].

Poor rates of clinic retention among adolescents also suggest that health system barriers exist to full commitment to HIV care [83]. Adolescent perceptions towards HIV medications are one of the most frequently reported barriers [84, 85], followed by fear of stigma and discrimination [86, 87]. This, along with reduced family supervision and transitions from peadiatric to adult care, could further limit follow-up as adolescents age [88].

Employment may be another explanation for the LTFU difference by age that we observe. The transition into adulthood may be associated with greater family responsibility for some adolescents, increasing demands on time, and relegating personal health to a secondary priority [89, 90]. Financial pressure may also lead to migration searching for employment, necessitating changing ART treatment providers. There is convincing evidence that a significant proportion of patients categorised as LTFU in SSA may have remained on ART but transferred to a different ART centre due to migration or other work-related reasons [91, 92]. In sum, the fear of stigma and inadvertent disclosure, the need to transfer care from paediatric to adult settings, and the high labour mobility of older adolescents may make them particularly prone to changing facilities and, therefore, to LTFU misclassification.

### Study limitations and future research

This systematic review and meta-analysis have limitations that should be considered in interpreting its findings. Firstly, the studies included in the review had different patient follow-up times, which could have resulted in the under-or over-estimation of the overall proportion of LTFU. Secondly, most of the studies included in this review had small sample sizes, which might have affected the proportion of LTFU reported in them. Another limitation of the studies we reviewed was a lack of reporting on critical contextual health system factors that might affect ART programs’ ability to follow up patients and deliver quality care, such as how long the program had been in operation or the quantity and training of program staff. A fourth limitation is that the review only included articles published in English, which may have led to the omission of studies from Francophone and Lusophone Africa. Some studies also consider older adolescents as adults in their report, making it difficult to consider the data.

Finally, most studies reviewed were conducted in only small parts of sub-Saharan African countries, most of which had well-established, mature national HIV/AIDS treatment programs. Therefore, the estimates of LTFU we found may not represent countries with newer, smaller HIV/AIDS treatment programs.

This study also has several strengths. These include a search strategy developed with a specialised librarian’s assistance, including articles from multiple databases and manuals (reference lists), electronic searches, and rigorous data abstraction and analysis.

The limitations related to the content of primary articles highlight the need for additional LTFU research with adolescent-specific outcomes, including interventional studies and qualitative research to explore treatment experience and reasons for LTFU.

### Policy implications

Our findings suggest the need for more finely grained, age-based HIV treatment protocols and counselling guides in the region. Current HIV treatment guidelines use age categories that largely ignore the unique needs of adolescents living with HIV. Patients less than 15 years of age are treated as paediatric patients and those 15 years and above as adults [93]. The development of supplemental guidelines and programs for ALHIV would address this life stage’s unique needs. For example, particular interventions could address young people’s fears about confidentiality and embarrassment about discussing health concerns, particularly reproductive health concerns, which form a powerful barrier to accessing care [94, 95]. Health professionals may benefit from guidance to overcome some of the challenges of working with young people, including communication difficulties, time constraints, uncertainty about the medico-legal status of those under 18 years, and managing consultations with parents present [96].

In addition to HIV-specific and clinical interventions, adolescents may require complementary services such as comprehensive, youth-friendly, family planning services and linkages to social protection and employment services. Supportive environments may be crucial for engaging adolescents living with HIV in their care. Programs that incentivise in-person follow-up with in-kind or cash benefits could be compelling in this population and improve older adolescents’ retention. There is evidence that these incentives can improve patient retention and reduce LTFU in adults [97, 98]. Finally, we recommend further qualitative studies to explore the client experience and possible reasons for LTFU among adolescents and additional interventional studies that assess programs to reduce LTFU in this population.

## Conclusions

This systematic review and meta-analysis revealed that LTFU among adolescents receiving ART in SSA is considerably higher than in other regions. Older adolescents in the region are at higher risk for LTFU than younger adolescents. Our results provide crucial baseline LTFU estimates that policymakers and stakeholders can use to evaluate services and treatment initiatives to improve HIV treatment retention.

## Supporting information

S1 File PRISMA checklist. (DOCX)

S2 File Searching strategy (Boolean operators and MeSH) terms). (DOCX)

S3 File Quality assessment. (DOCX)

S4 File Quality assessment detail. (DOCX)
