# Supplementary material for: The proportion of loss to follow-up from antiretroviral therapy (ART) and its association with age among adolescents living with HIV in sub-Saharan Africa: A systematic review and meta-analysis
Source: PLoS One. 2022 Aug 11;17(8):e0272906. doi: 10.1371/journal.pone.0272906 (PMC9371308; doi:10.1371/journal.pone.0272906)
Supplement: S2 File — (DOCX) [file pone.0272906.s002.docx]

|  | **Concept 1**  **lost to follow-up** | **Concept 2**  **ART** | **Concept 3**  **adolescents** | **Concept 4**  **HIV** | **Concept 5**  **Sub-Saharan Africa** |
| --- | --- | --- | --- | --- | --- |
| **Subject headings** | MeSH: lost to follow-up  Emtree: follow up  CINAHL MeSH:  DE: | **MeSH**: Anti-Retroviral Agents  **MeSH**: Anti-HIV Agents  **Emtree**: antiretroviral therapy  **CINAHL MeSH**: Anti-Retroviral Agents  **DE**: n/a | **MeSH**: Adolescent  **Emtree**: Adolescent  CINAHL MeSH:  DE: | **MeSH**:HIV  **Emtree**: Human immunodeficiency virus  **CINAHL** MeSH: [Human Immunodeficiency Virus](javascript:XslPostBack('ctl00$ctl00$MainContentArea$MainContentArea$ctrlResults','meshList','index%7C1%24term%7CHuman%20Immunodeficiency%20Virus%24cmd%7CmeshList');)  **DE**:HIV | **MeSH**: Africa South of the Sahara  Emtree: Sub-Saharan Africa  CINAHL MeSH: Subsaharan Africa  DE: Africa, Sub-Saharan |
| **Keywords** | retention | Anti-retroviral therapy OR antiretroviral therapy | Teen  youth  young adults  child  adolescen | HIV  Human  immunodeficiency virus  AIDS  AIDS Virus  Acquired Immune Deficiency Syndrome Virus  HIV infections  Immunodeficiency Virus | List all countries  Angola, Benin, Botswana, Burkina Faso, Burundi, Cameroon, Cape Verde, Central African Republic, Chad, Comoros, Congo, Democratic Republic of Congo, Cote d'Ivoire, Djibouti, Equatorial Guinea, Eritrea, Ethiopia, Gabon, Gambia, Ghana, Guinea, Guinea-Bissau, Kenya, Lesotho, Liberia, Madagascar, Malawi, Mali, Mauritania, Mauritius, Mozambique, Namibia, Niger, Nigeria, Réunion, Rwanda, Sao Tome and Principe, Senegal, Seychelles, Sierra Leone, Somalia, South Africa, Sudan, Swaziland, Tanzania, Togo, Uganda, Western Sahara, Zambia, Zimbabwe |
|  | treatment failure | Antiretroviral drugs OR anti-retroviral drugs |  |  |  |
|  | loss to follow-up | ART |  |  |  |
|  | lost to follow-up | AIDS drugs |  |  |  |
|  | loss to follow up | Anti-AIDS agents |  |  |  |
|  | treatment outcome | Anti-AIDS drugs |  |  |  |
|  | lost to follow up | Anti-HIV drugs |  |  |  |
|  | LTFU |  |  |  |  |
|  |  |  |  |  |  |
|  |  |  |  |  |  |
|  |  |  |  |  |  |
