# Supplementary material for: The proportion of loss to follow-up from antiretroviral therapy (ART) and its association with age among adolescents living with HIV in sub-Saharan Africa: A systematic review and meta-analysis
Source: PLoS One. 2022 Aug 11;17(8):e0272906. doi: 10.1371/journal.pone.0272906 (PMC9371308; doi:10.1371/journal.pone.0272906)
Supplement: S3 File — (DOCX) [file pone.0272906.s003.docx]

| **Articles** | **Selection 1)**  **a=b=1, c=d=0** | **Selection 2)**  **a=1, b=0** | **Selection 3)**  **a=1, b=0, c=0** | **Selection 4)**  **a=2, b=1, c=0** | **Comparability 1) a=b=1** | **Outcome 1)**  **a=b=2, c=1, d=0** | **Outcome 2)**  **a=1, b=0** | **Score** |
| --- | --- | --- | --- | --- | --- | --- | --- | --- |
| Bakanda et al | a) | a) | a) | c) | a) b) | b) | a) | 8 |
| Arrlve et al | b) | b) | a) | a) | a)b) | c) | b) | 7 |
| Bygrave et al | b) | a) | b) | b) | a) b) | a) | a) | 8 |
| Nglazle et al | b) | b) | b) | b) | a) b) | d) | a) | 5 |
| Shroufl et al | c) | a) | c) | b) | a) b) | c) | a) | 6 |
| Evans et al | a) | a) | b) | b) | a) b) | c) | a) | 7 |
| Merkel et al | b) | b) | a) | a) | a) b) | c) | a) | 8 |
| Marry-Ann et al | a) | a) | a) | c) | a) b) | b) | a) | 5 |
| Ojikutu et al | b) | b) | a) | a) | a)b) | c) | b) | 7 |
| Nabukeera-Barungi et al | b) | a) | b) | b) | a) b) | a) | b) | 7 |
| Nsanzlmana et al | b) | b) | b) | b) | a) b) | d) | a) | 5 |
| Matyanga et al | c) | a) | c) | b) | a) b) | c) | a) | 7 |
| Koach et al | a) | a) | b) | b) | a) b) | c) | a) | 7 |
| Okobol et al | a) | a) | a) | a) | a) b) | c) | a) | 9 |
| Fwemba et al | a) | a) | a) | b) | a) b) | b) | a) | 9 |
| Kranzer et al | a) | a) | a) | b) | a) b) | b) | a) | 9 |
| MacKenzie et al | a) | a) | b) | a) | a)b) | b) | a) | 8 |
| McHugh et al | b) | a) | b） | a) | a) b) | b) | a) | 8 |
| Vogt et al | a) | a) | a) | a) | a) b) | b) | b) | 9 |
| Schomaker et al | a) | a) | a) | a) | a) b) | d) | a) | 8 |
| Fatti et al | a) | a) | b) | a) | a) b) | b) | a) | 9 |
| Kariminia et al | a) | a) | a) | a) | a) b) | d) | a) | 8 |
| Slogrove | b) | a) | b) | a) | a)b) | b) | a) | 8 |
| Slogrove AL et al | a) | b) | a) | b) | a) b) | b) | a) | 8 |
| Anderson et al | c) | a) | a) | c) | a) b) | a) | a) | 7 |
| Jerene et al | a) | a) | a) | c) | a) b) | c) | a) | 7 |
| Ngeno et al | a) | a) | a) | c) | a) b) | b) | a) | 8 |
| Tsondal et al | a) | a) | a) | c) | a) b) | b) | a) | 8 |
| Munyayi et al | b) | a) | a) | c) | a) b) | b) | a) | 8 |

NB: high-quality score > 6, NOS quality assessment
