# Supplementary material for: The proportion of loss to follow-up from antiretroviral therapy (ART) and its association with age among adolescents living with HIV in sub-Saharan Africa: A systematic review and meta-analysis
Source: PLoS One. 2022 Aug 11;17(8):e0272906. doi: 10.1371/journal.pone.0272906 (PMC9371308; doi:10.1371/journal.pone.0272906)
Supplement: S4 File — (DOCX) [file pone.0272906.s004.docx]

| Studies |  | Status |
| --- | --- | --- |
| Bakanda et al | - The sample size is not determined using the recommended assumptions - Response rate (i.e., the proportion of the study sample completing the study and providing outcome data) is not adequate. - Quality management is not adequately described, no training for data collectors given | High |
| Arrive et al | - Blinding issues for the two group is not well monitored | High |
| Bygrave et al | - The sample size is not determined using the recommended assumptions - Response rate (i.e., the proportion of the study sample completing the study and providing outcome data) is not adequate. - Quality management is not adequately described, no training for data collectors given - The study not applied the proper analytical method, it is not clear how interesting outcome was computed and did not control the confounder | High |
| Nglazi et al | - Response rate (i.e., the proportion of the study sample completing the study and providing outcome data) is not adequate. | High |
| Shroufi et al | - The sampling frame and recruitment are not adequately described, - Inclusion and exclusion criteria are not adequately described - The type of analysis was not clearly stated Variables are not clearly described. - All essential confounders, including treatments (critical variables in the conceptual model), are not measured. - There is no clear definition of the outcome. - The selected model is not adequate for the design of the study. | Low |
| Evans et al | - Follow-up time is not clearly described - Interesting variables are not clearly stated - The measurement tool is not checked for quality | High |
| Merkel et al | - Inclusion and exclusion criteria are not adequately described - Response rate (i.e., the proportion of the study sample completing the study and providing outcome data) is not adequate. - Quality management is not adequately described, no training for data collectors given - The study not applied the proper analytical method, it is not clear how interesting outcome was computed and did not control the confounder | High |
| Mary-Ann Davies et al |  | Low |
| Ojikutu et al | - Study sites selected using a non-probability sampling method - The sample size is not clearly determined - Variable of the interesting outcome, lost to follow-up, is not clear, operationally, described - Inclusion and exclusion criteria are not adequately described - Response rate (i.e., the proportion of the study sample completing the study and providing outcome data) is not adequate. | High |
| Nabukeera-Barungi et al | - The analysis is not clearly described. - The confounding variables are not controlled - The model assumption is not checked - The interesting outcome is not clearly defined - The sampling frame and recruitment is not adequately described, | High |
| Nsanzimana, et al | - The sample size is not determined using the recommended assumptions - Response rate (i.e., the proportion of the study sample completing the study and providing outcome data) is not adequate. - Quality management is not adequately described, no training for data collectors given | High |
| Matyanga et al | - Small sample size (number adolescents is not sufficient to explain the outcome) - A low proportion of the participants were included in the study - No information on response rate | - High   High |
| Koech et al | - Inclusion and exclusion criteria are not adequately described - A clear definition of the outcome of interest is not clearly provided, including duration of follow-up and - The outcome measure and method used are adequately valid and reliable to limit misclassification bias is not described - The model assumption is not checked for the analysis | High |
| Okoboi et al | - Eligibility criteria are not clearly defined - An interesting variable is not clearly defined | High |
| Vogt et al | - The study sample size is not adequately described for key characteristics - The source population or population of interest is not adequately described for key characteristics. The sampling frame and recruitment are not adequately described inclusion and exclusion criteria are not adequately described Variable of the study not clearly described An interesting outcome is not clearly defined The model assumption for the analysis is not checked The analysis does not consider the confounding effect The quality of the measurement tool is not checked The study experienced a high non-response rate, which may bias the finding | Low |
| Fwemba et al | - Study variables are not clearly listed - The analysis is not specified, described | High |
| McHugh et al | - The sample size is not included - The outcome variable is not defined - The analysis is not detailed well | High |
| Kranzer K., et al | - The source population or population of interest is not adequately described for key characteristics. - The follow-up period is not clearly described - Interesting outcome is not clearly defined - The sampling frame and recruitment is not adequately described, | High |
| Schomaker et al | - Population group is not clear - The proportion of the group is not well indicated | Low |
| MacKenzie et al | - The outcome measure and method used are adequately valid and reliable to limit misclassification | High |
| Slogrove et al | - The sampling frame and recruitment are not adequately described - Variable of interest is not clearly described - The study outcome is not clearly defined - The eligibility criteria are not adequately described - The analysis is not clear - All important confounders are not measured. - Type of measuring tool is not described - The analysis does not control the confounder | High |
| Kariminia et al | - The outcome variable is not clearly defined. - A different group of the population described in the method sections | High |
| Fatti et al | - Variable of the interesting outcome, lost to follow-up, is not clear, operationally, described | High |
| Slogrove AL et al | - The follow-up period is short The sampling frame and recruitment are not adequately described Variable of the interesting outcome, lost to follow-up, is not clearly described The eligibility criteria are not adequately described The analysis is not clear All-important confounders are not measured. Type of measuring tool is not described, cleared The analysis does not control the confounder | High |
| Anderson et al | - The sampling frame and recruitment are not adequately described - Variable of the interesting outcome, lost to follow-up, is not clearly described - Quality management is not adequately described, no training for data collectors given - The analysis is not precise and does not control the confounder - Type of measuring tool is not described, cleared - No information on response rate | High |
| Ngeno et al | Source population is not clearly defined  The sample size is not computed using recommended assumptions | High |
| Jerene et al | The standard tool is not used to measure the outcome. | High |
| Tsondai et al | Unclear | Low |
| Munyayi et al | The study population is not clearly defined (children/adolescent)  The model assumption for the analysis was not checked  The analysis not considered controlling the confounding Variable | High |
